# Heterogeneous Response of Airway Eosinophilia to Anti-IL-5 Biologics in Severe Asthma Patients

**DOI:** 10.3390/jpm12010070

**Published:** 2022-01-07

**Authors:** Maruša Kopač Šokić, Matija Rijavec, Peter Korošec, Urška Bidovec-Stojkovič, Izidor Kern, Romana Vantur, Sabina Škrgat

**Affiliations:** 1 University Clinic of Respiratory and Allergic Diseases Golnik, 4204 Golnik, Slovenia; kopac.marusa@gmail.com (M.K.Š.); matija.rijavec@klinika-golnik.si (M.R.); peter.korosec@klinika-golnik.si (P.K.); Urska.Bidovec-Stojkovic@klinika-golnik.si (U.B.-S.); izidor.kern@klinika-golnik.si (I.K.); romana.vantur@klinika-golnik.si (R.V.); 2 Biotechnical Faculty, University of Ljubljana, 1000 Ljubljana, Slovenia; 3 Faculty of Pharmacy, University of Ljubljana, 1000 Ljubljana, Slovenia; 4 Department of Pulmonology, Division of Internal Medicine, University Medical Centre Ljubljana, 1000 Ljubljana, Slovenia; 5 Faculty of Medicine, University of Ljubljana, 1000 Ljubljana, Slovenia

**Keywords:** severe asthma, anti-IL-5 biologics, induced sputum, airway eosinophilia

## Abstract

Many questions concerning responders (R) and nonresponders (NR) in severe eosinophilic asthma (SEA) after blocking the IL-5 (interleukin 5) pathway are still not clear, especially regarding the early parameters of response to biologics in personalized treatment strategies. We evaluated 17 SEA patients treated with anti-IL-5 biologics (16 patients mepolizumab, one patient benralizumab) before the introduction of biologics, and at a week 16 follow-up. Clinical, cellular and immunological parameters in peripheral blood were measured in R and NR. Sputum induction with the measurement of cellular and immunological parameters was performed at 16 weeks only. There were 12 R and 5 NR to biologics. After 16 weeks, there was a significant improvement in percentages of FEV1 (*p* = 0.001), and asthma control test (ACT) (*p* = 0.001) in the R group, but not in NR. After 16 weeks, the eosinophils in induced sputum were 27.0% in NR and 4.5% in R (*p* = 0.05), with no difference in IL-5 concentrations (*p* = 0.743). Peripheral eosinophilia decreased significantly in NR (*p* = 0.032) and R (*p* = 0.002). In patients with SEA on anti-IL-5 therapy, there was a marked difference in airway eosinophilic inflammation between R and NR already at 16 weeks, after anti-IL-5 introduction.

## 1. Introduction

Severe asthma is a debilitating disease associated with persistent symptoms, poor quality of life, frequent use of oral corticosteroids (OCS), increased hospitalization rates, and detrimental side effects of OCS [1,2,3]. The treatment of asthma is moving toward a personalized treatment strategy based on patient-specific characteristics and underlying endotypes rather than disease severity alone [4].

A subset of patients with moderate to severe asthma have an eosinophilic phenotype characterized by an increase in sputum and/or blood eosinophils, despite treatment with corticosteroids, and are more prone to frequent exacerbations [4,5,6,7]. However, though cross-sectional data corroborates the statement that T2 inflammation is found in around 50% of patients with severe asthma [8], recent findings in a real-life difficult-to-treat UK asthma population have demonstrated that, in fact, the vast majority (83%) of difficult-to-treat asthma patients have evidence of eosinophilia, defined as ≥300 cells/μL on at least one occasion in the last 10 years [9]. Interleukin 5 (IL-5) is the primary cytokine involved in the recruitment, activation, and survival of eosinophils, and by inhibiting this pathway, anti-IL-5 biologics reduce eosinophilic airway inflammation [10]. It is well known that the response to anti-IL-5 biologics is not equal in every patient. Some patients are responders and can reach complete asthma control, or are responders that still experience some residual disease manifestations. On the other side nonresponders show no improvement, or have clinical worsening [11,12]. The underlying mechanisms of these individual different responses still need additional clarification.

Several studies have looked at predictions for the response to anti-IL-5 treatment, in which response was mostly defined through the reduction of exacerbations or OCS use. Higher eosinophil counts or higher exacerbation rates seem to be the best predictors of positive responses to anti-IL-5 treatment [13,14,15].

Nevertheless, predicting responses to biologics therapy remains problematic. The assessment of inflammatory parameters at the level of the airway in induced sputum represents one of the additional personalised approaches in asthma endotyping. Indeed, the presence of a high sputum eosinophil count was found to be predictive of a response to corticosteroid therapy [16]. The assessment of eosinophil counts in the blood as a biomarker is, on the other hand, a procedure that is available in every day clinical practice. However, data are showing that although absolute blood eosinophil count correlates with airway (sputum) eosinophil numbers in patients who are on low to moderate doses of ICS [17], there is a lack of concordance in those on maintenance OCS [18].

Patients with severe eosinophilic asthma have an exaggerated eosinophilopoeitic process in their airways, and treatment with biologics has shown a heterogeneous response of airway eosinophil count to anti-IL-5 therapy [19,20,21]. According to a very recent publication on real-life long-term therapy response to anti-IL-5 biologics in severe asthma, a super-response was observed in 14% of patients. It was predicted by shorter asthma duration, higher FEV1, and tended to be associated with adult-onset asthma, the absence of nasal polyps and lower BMI [22].

Many questions concerning responders and nonresponders after blocking the IL-5 pathway are still not clear, especially regarding the early signs and parameters of response to biologics. The aims of the present study were first to assess blood and induced sputum eosinophil counts in responders and nonresponders, before and after 16 weeks of anti-IL-5 treatment; second, to evaluate the blood and induced sputum IL-5 concentrations; third, to evaluate clinical characteristics between the two groups.

## 2. Materials and Methods

### 2.1. Study Subjects

We included 17 adult patients with severe asthma visiting the outpatient clinic at the University Clinic of Respiratory and Allergic Diseases Golnik, Slovenia. Patients met the criteria for the diagnosis of severe asthma according to ERS/ATS guideline criteria [23] and were treated with anti-IL-5 biologics (16 patients on mepolizumab 100 mg subcutaneously every 4 weeks, and one patient on benralizumab; 30 mg subcutaneously every 4 weeks for first 3 doses and then every 8 weeks) in the period between March 2017 and August 2019. At week 16 of follow-up, patients were classified as responders or non-responders to anti-IL-5 biologics. The response was defined as no exacerbation and/or discontinuation or reduction of the methylprednisolone dose ≥50% at the week 16 follow-up. Nonresponders discontinued their anti-IL-5 biologic. Responders continued with their anti-IL-5 biologic and the number of exacerbations in the last year with the need for bursts of OCS treatment was recorded after 12 months of follow-up. The exclusion criteria included COPD and active smoking.

At baseline and the week 16 follow-up, clinical parameters (demographics, asthma duration, smoking history, comorbidities, hypersensitivity to Aspirin and NSAIDs, chronic rhinosinusitis, allergies), presence of gastroesophageal reflux disease (GERD), nasal polyposis, bronchiectasis, nitric oxide in exhaled air (FeNO), prebronchodilator forced expiratory volume in 1 s (FEV1), an asthma control test (ACT), cellular (absolute amount of eosinophils in peripheral blood) and immunological (IL-5 concentration in peripheral blood) were measured in responders and nonresponders. Sputum induction was performed at 16 weeks only. Therefore, the percentage of eosinophils in the induced sputum and IL-5 concentration in the induced sputum were determined at 16 weeks only.

Data were derived from electronic patient files.

All patients provided written informed consent and the study was performed with the approval of the national review board no. 0120-263/2019/4.

### 2.2. Measurements

#### 2.2.1. Lung Function Tests

Spirometry was carried out according to American Thoracic Society Criteria [24] on a spirometer (Vyntus CPX, CareFusion Germany 234 GmbH, Hochberg, Germany). FeNO was measured with the online chemiluminescence FeNO analyser CLD 88 Series (ECOMEDICS, Duernten, Switzerland) according to published guidelines from the European Respiratory Society (ERS) and the American Thoracic Society (ATS) [25].

#### 2.2.2. Induced Sputum Analysis

Subjects initially inhaled 0.9% NaCl solution for 30 s, 1 min, and 5 min via an ultrasonic nebulizer (PARI MASTER Type 84.0100, PARI GmbH, Starnberg, Germany). If no sputum was obtained, subjects continued to inhale 3% hypertonic NaCl solution for 8.5 min, and 4.5% hypertonic NaCl solution for 15.5 min. At least 2 mL of sputum was collected into a sterile container. In the cytology laboratory, the sputum was immediately processed and homogenized with 0.1% dithiothreitol (Sputolysin, Calbiochem, San Diego, CA, USA), and cell-free supernatants were frozen at −80 °C until subsequent analysis. The total number of non-squamous cells (TNNC) per ml of sputum sample was assessed using a hemocytometer. Cytospins were stained according to the May–Grünwald–Giemsa and Papanicolaou methods. Differential cell counts were performed by one observer counting 200 non-epithelial cells. The quality of the induced sputum was assessed according to the recommendations of Pizzichini E et al. [26], and only samples with a score of 7 or more were used for further analysis.

#### 2.2.3. IL-5 Measurement

The enzyme-linked immunosorbent assay (ELISA) was performed with a Quantikine^®^ test (R&D Systems, Minneapolis) to detect IL-5 in the cell supernatants and the sera of the subjects. Briefly, microplates with monoclonal antibodies specific for human IL-5 were incubated with standards, controls and samples (serum or induced sputum supernatant), followed by a wash and incubation with enzymatically bound monoclonal antibodies to form sandwich complexes. After incubation and a wash, the substrate solution was added, and the plate was re-incubated. Depending on the amount of IL-5 the microplate pits dyed to different fluorescence intensities. The absorbance was measured spectrophotometrically. A calibration curve was created, and the concentration of IL-5 was calculated using a graph of the obtained formula.

#### 2.2.4. Statistical Analysis

Differences in parameters before and after the introduction of mepolizumab within each group (responders, nonresponders) were analysed by using the Wilcoxon *t*-test. We used the Mann–Whitney *t*-test to compare the same parameter between both groups at baseline and the week 16 follow-up. Differences were considered significant if *p*-values were <0.05. Confidence interval: 95%. The statistical program GraphPad Prism 8.0.1 was used to perform the statistical analysis and the presentation of the results.

## 3. Results

### 3.1. Study Subjects

Data from 17 adult patients with severe asthma and treatment with anti-IL-5 biologics were analysed on the day before the first administration, and 16 weeks after the introduction of the drug. Twelve [70.6%] patients met the definition of a responder and five [29.4%] were nonresponders. The patient treated with benralizumab was a responder.

Basic characteristics, asthma treatment parameters, pulmonary function test parameters and inflammatory markers at baseline and follow-up at 16 weeks of both groups are shown in Table 1 and Table 2.

### 3.2. Basic Characteristics and Asthma Treatment

There were no differences in age (*p* = 0.339), asthma duration (*p* = 0.200) and ACT (*p* = 0.795) at baseline between responders and nonresponders. Nobody was a current smoker. The history of smoking was higher in responders (*p* = 0.280). All patients were on inhaled glucocorticoid therapy with a median of 600 mcg in beclomethasone equivalent and with no statistical difference at baseline. All patients who were nonresponders needed oral glucocorticoid maintenance therapy at baseline, compared with responders where only one quarter of patients needed oral glucocorticoid treatment. There were no differences in nasal polyposis, but there was a trend toward a higher frequency of GERD, rhinosinusitis and NSAID intolerance in responders. These differences did not reach a statistical significance between the two groups (Table 1).

### 3.3. Glucocorticoid Load

Following the introduction of mepolizumab, all five nonresponders continued with oral glucocorticoid therapy, compared with the responders who discontinued OGC completely (Table 2). There was a trend toward lowering the median daily dose of methylprednisolone before administration in nonresponders from 12 mg (IQR 5–20) of methylprednisolone at baseline, to 8 mg (IQR 5–10) of methylprednisolone daily, after the administration of anti-IL-5 (*p* = 0.468). ICS doses (converted to beclomethasone equivalents) were not statistically significantly reduced in any group during the 16 weeks of biologic treatment. Details are shown in Table 2.

### 3.4. Pulmonary Function Test

There was a trend toward better responders’ median for prebronchodilator FEV1 as absolute values and as percentages of predicted values compared with nonresponders at baseline, but there was no statistically important difference between the two groups (*p* = 0.234) (Table 1). In the responders’ group, there was a statistically significant improvement in FEV1 as absolute values and as percentages of predicted values after 16 weeks of anti-IL-5 therapy. In nonresponders, this difference was statistically insignificant in both millilitres and percentages, as displayed in Table 2.

### 3.5. Quality of Life and Exacerbation Rate

Quality of life and disease control were measured with the ACT questionnaire. There was a positive trend in ACT scoring in both groups after the anti-IL-5 introduction, but a statistically significant improvement was found only in responders (*p* = 0.001).

All 17 subjects had exacerbations in the year prior to anti-IL-5 treatment, requiring bursts of OCS, and there was no statistical difference (*p* = 0.556) at baseline between the number of exacerbations/year with the need of oral glucocorticoid therapy between responders and nonresponders. The median number of exacerbations in responders in the year before the anti-IL-5 introduction was 3.5 (IQR 2.3–4.0) and the one year follow up after the introduction was 0 (IQR 0–0). The number decreased statistically significantly (*p* < 0.0001) in responders (Table 2).

The median of exacerbations in non-responders was 5 (IQR 2.5–5.5) in the year before biologics introduction. The drug was discontinued after 16 weeks due to insufficient clinical response.

### 3.6. Inflammatory Markers

#### 3.6.1. Blood Eosinophils

**Figure 1:**
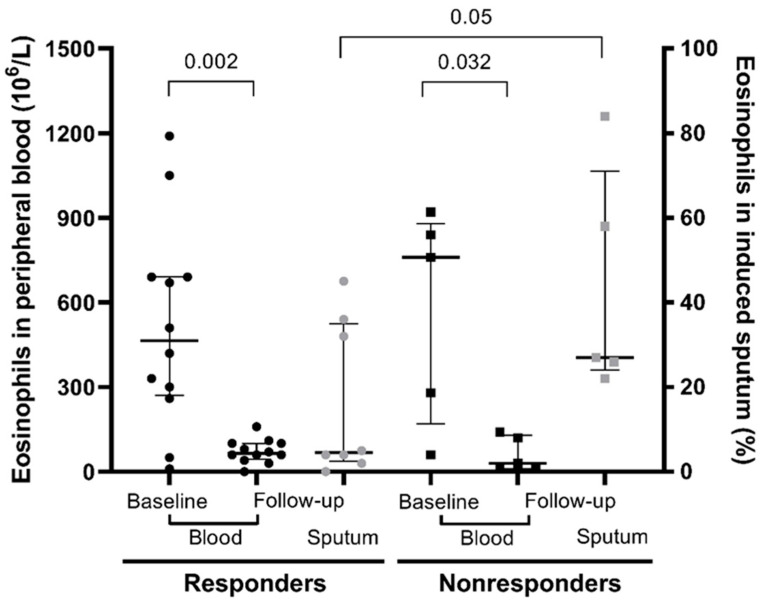
Differences in blood and sputum eosinophils between the two groups at baseline and at the week 16 follow-up.

Absolute values of eosinophils in the patients’ sera were evaluated. The difference in baseline values between the two groups was not statistically significant (*p* = 0.629). Peripheral eosinophilia decreased statistically significantly in both groups with the introduction of biological therapy (nonresponders *p* = 0.032; responders *p* = 0.002). The difference in the level of peripheral eosinophilia on target therapy between the two groups was not statistically significant (*p* = 0.662), and is shown in Figure 1.

#### 3.6.2. Eosinophils in Induced Sputum

Significant differences were found in the level of airway eosinophilia (Figure 1). According to the criteria of the cytology laboratory [24], four samples were not representative (all of them from the group of responders). The median percentage of eosinophils in induced sputum on anti-IL-5 therapy was 27.0% in nonresponders (IQR 24–71), and 4.5% in representative responder samples (IQR 2.5–35) after 16 weeks of anti-IL-5 therapy.

### 3.7. IL-5 in the Blood and Sputum

**Figure 2:**
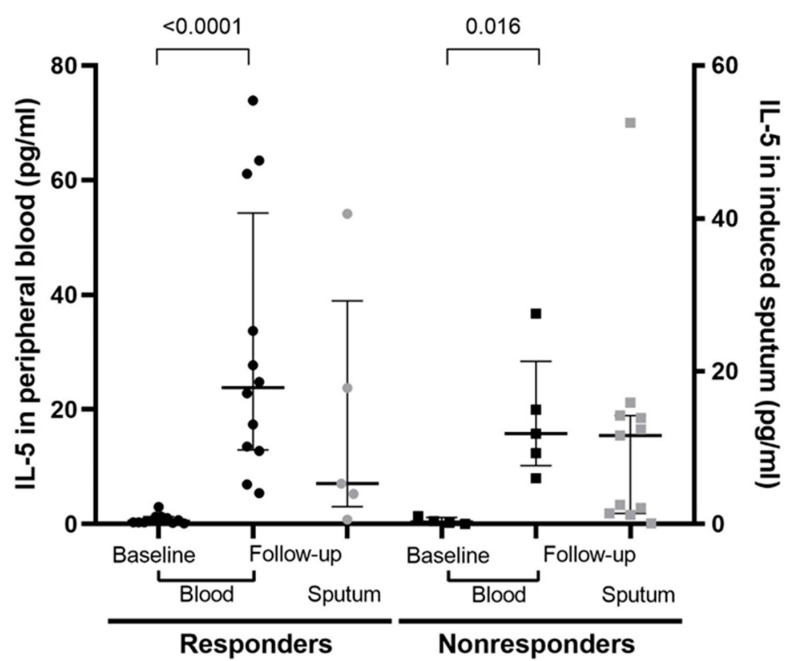
IL-5 concentrations in blood and sputum in both groups at baseline and follow-up.

The immunological marker IL-5 in serum was evaluated. Baseline and follow-up values were not statistically significant between responders and nonresponders (before introduction *p* = 0.563, after introduction *p* = 0.442). We found a statistically significant increase in serum IL-5 at 16 weeks after the initiation of treatment, compared with the pre-treatment values in responders and nonresponders (*p* = <0.0001 and *p* = 0.016, respectively). Details are illustrated in Figure 2.

We also analysed IL-5 concentrations in 16 induced sputum samples taken during treatment only. No statistically significant differences were found between responders and nonresponders after the introduction of mepolizumab (*p* = 0.743).

In 13 (76.4%) subjects, FeNO was not measured after the introduction, so this parameter was not included in further analysis.

## 4. Discussion

In the present study on patients with severe eosinophilic asthma on anti-IL-5 therapy, we showed that a marked difference already exists in airway eosinophilic inflammation between responders and nonresponders at 16 weeks after anti-IL-5 introduction. Nonresponders with a median of 27% sputum eosinophils in induced sputum had six-fold more sputum eosinophils at follow-up than responders. Moreover, all responders completely stopped with their OCS maintenance therapy, and all of the patients who were nonresponders had to continue with their OCS due to uncontrolled asthma, despite anti-IL-5 treatment. At the same time, there was no impact of ICS, as there was no diminution of ICS dosing compared to baseline treatment in responders and nonresponders.

According to a very recent real-life publication of Bel group [22], our responders mostly behaved as super-responders, and they had a convenient airway eosinophilic status compared with nonresponders, 16 weeks after anti-IL-5 introduction. Clinically, there was concordance, as we also showed a statistically significant improvement in lung function and subjective condition (ACT score) in responders only, which is in concordance with past clinical studies [21,27,28].

Despite persistent eosinophilic load in nonresponders, there was a clear reduction of peripheral eosinophilia. This reduction was nearly the same when compared with responders. This local persistent eosinophilic activity may be the cause of symptom persistence and exacerbations in nonresponders. Discordance between the systemic versus luminal anti-eosinophil effect of anti-IL-5 therapy was indicative of the alternative mechanisms of in situ eosinophilic inflammation, which, when unsuppressed, may contribute to ongoing clinical symptoms [19,29].

This heterogeneity of the response to anti-IL-5 treatments might be a consequence of several additional factors, such as individual differences in pharmacokinetics and actual plasma drug levels [15], or a consequence of the monoclonal antibody induction of immunologic response with the formation of anti-drug antibodies and a secondary loss of response [30,31]. A significant number of patients who met the currently approved indications for anti-IL5 mAbs showed suboptimal responses to them in real-life clinical practice, particularly if they were on high doses of prednisone [32]. All nonresponders from our study group were OCS dependent, with a median daily methylprednisolone of 12 mg, indicating that there was a significant difference in OCS load between responders and nonresponders before the introduction of biologics.

Possible residual disease manifestations may result from the ongoing activation of non-IL-5 driven inflammatory pathways, such as the IL-4/IL-13 [33,34,35]. In this case of persistency of airway eosinophilia, it would be appropriate to consider a change of biologics toward blocking the IL-4/IL-13 pathways. Furthermore, some patients with severe asthma may have mixed eosinophilic and neutrophilic inflammation that does not respond well to the biologic that targets only one part of the inflammatory pathway [36,37].

We are aware that sputum induction at 16 weeks, on top of the clinical evaluation, seems early in the algorithm after the biologic introduction, since treatment duration for the initial 12 months is recommended due to the possibility of delayed treatment responses [38]. In the cohort of Drick et al., however, they did not observe any delayed treatment response in patients who failed to respond early after therapy initiation. In regard to high treatment costs, regular assessment seems mandatory to detect treatment nonresponders early [39], and the assessment of eosinophilic load at the level of the airway seems to be of added value.

In case of treatment failure, comorbidities should be taken into consideration. Only a few studies have been conducted on potential clinical characteristics to help define the therapy response [22]. Our study did not find a statistically significant difference in smoking history, the presence of GERD, chronic rhinosinusitis, nasal polyposis nor NSAID hypersensitivity, and this might be a consequence of the small number of patients in responders and nonresponders. In a previous study, despite the small number of patients, they carefully suggested that the profile of a true super-responder to long-term anti-IL-5 biologics was an adult with a relatively short duration of eosinophilic asthma, without nasal polyps, chronic airflow limitation, or overweight. Further research in larger cohorts is needed to confirm these findings [22].

We did not confirm statistically significant differences in IL-5 concentrations before and after the introduction of biologic therapy, either in blood or induced sputum. We found a statistically significant increase in IL-5 after the initiation of treatment compared with the baseline values in nonresponders and responders. This has also been observed in previous studies [40,41,42]. Possible causes of elevated IL-5 levels during anti-IL5 treatment are the formation of immune complexes between IL-5 and anti-IL-5 antibodies (monoclonal antibodies bind IL-5 and prevent degradation of IL-5), increased IL-5 receptor expression, and/or increased T helper cells that produce intracellular IL-5. To date, evidence for these hypotheses is lacking [4,30,43]. The main cause of the formation of immune complexes is thought to be insufficient to reach into the tissue with the biologic drug. However, IL-5 elevations have also been observed with higher doses of mepolizumab (750 mg) and with other “hypereosinophilic” diseases, such as hypereosinophilic syndrome and eosinophilic esophagitis [4,40]. It is therefore not concluded whether certain eosinophils are unresponsive to anti-IL-5 treatment, or whether the problem is an under-dosing of the biologic. Additionally, other cytokines, or even other cells (e.g., neutrophils), may be involved in eosinophil activation that cannot be influenced by anti-IL-5-specific therapy, or eosinophil synthesis may take place outside of the bone marrow. This indicates that the pathogenesis of T2 inflammation is complex, and that anti-IL-5 therapy acts only on part of the complex pathways [4,40,43]. As a result, neither serum nor sputum IL-5 concentrations have been shown to be a successful biomarker for predicting responses to therapy, and this finding may also be due to the small number of samples.

The limitation of this study was the lack of induced sputum data before the introduction of anti-IL-5, as this would serve as a reference for the individual patient, and it would enable us to follow the dynamic of eosinophilia from the biologic naive airway toward early examination at 16 weeks of treatment.

Another limitation of this small real-world study was the incompleteness of the data concerning NO measurement and BMI (body mass index). The additional value would represent data concerning the number/percentage of neutrophils in induced sputum that would give us an additional view of the inflammation profile in the asthmatic airway.

## 5. Conclusions

In the present study on patients with severe eosinophilic asthma on anti-IL-5 therapy, we showed that there a marked difference exists in airway eosinophilic inflammation between responders and nonresponders at 16 weeks after anti-IL-5 introduction. Non-responders with a median of 27% sputum eosinophils in induced sputum had six-fold more sputum eosinophils at follow-up than responders. In patients who were responders, we characterised improvements in lung function, the number of exacerbations, glucocorticoid burden and subjective (ACT) clinical parameters, whereas in nonresponders these did not improve, or the improvement was not statistically significant.

We are aware that induced sputum is not an easily obtainable sample as the procedure is demanding and linked to few centres. According to the results of this small real-life study we propose that it is worth considering an induced sputum analysis in patients with unsatisfactory or limited response to biologic. We recommend that these interesting preliminary findings will need validation in future larger studies to confirm our results.

## Figures and Tables

**Table 1:** Baseline characteristics of subjects.

Basic Characteristics	Baseline Responders	Baseline Nonresponders	*p*-Value
Sex, male	2	1	0.879
Sex, female	10	4	
Age in years, median (IQR)	59 (49.5–71.8)	53 [44–64)	0.338
Smoking history—n (%)	6 (50)	1 [20)	0.280
Smoking history—years, n (%)	1.5 (0–10)	0 (0–4)	0.266
Asthma duration—years, median (IQR)	11 (5.3–17.3)	14 (12.5–26)	0.200
ACT-median (IQR)	15 (8.3–19)	13.5 (7.3–19.8)	0.795
**Comorbidities**			
Nasal polyps—n (%)	5 (42)	4 (80)	0.294
GERD—n (%)	8 (67)	4 (80)	>0.999
Rhinosinusitis—n (%)	5 (42)	2 (40)	>0.999
NSAID intolerance—n (%)	7 (58)	2 (50)	0.619
**Asthma treatment**			
Regular ICS treatment-n (%)	12 (100)	5 (100)	0.246
ICS/day (mcg in beclomethasone equivalent)-median (IQR)	600 (450–1050)	600 (440–620)	0.296
OCS maintenance dose—n (%)	3 (25)	5 (100)	0.009
OCS maintenance dose (mg/day)—median (IQR)	0 (0–1.5)	12 (5–20)	0.001
OCS bursts due to asthma exacerbation—median (IQR)	3.5 (2.3–4)	5 (2.5–5.5)	0.556
**Pulmonary function test**			
FEV1 preBD (mL)—median (IQR)	1770 (1570−2040)	1330 (1010−2265)	0.234
FEV1 preBD (%)—median (IQR)	67.5 (67−80)	46 (36−68.5)	0.039
**Inflammatory markers**			
FeNO in exhaled air—median (IQR)	na	na	/
FeNO in exhaled air > 20 ppb, n (%)	4 (80)	11 (100)	0.313
Eosinophils in peripheral blood cells (10^6^/L)—median (IQR)	465 (270–690)	760 (170–880)	0.629
Eosinophils in induced sputum (%)	na	na	/
IL-5 in peripheral blood (pg/mL)—median (IQR) *	0.5 (0.3–1.3)	0.35 (0.05–1.2)	0.563
IL-5 in induced sputum (pg/mL)—median (IQR)	na	na	0.743

* Only sera of 11 responders and 4 nonresponders were included. Abbreviations: ACT, asthma control test; FeNO, nitric oxide in exhaled air; FEV1, forced expiratory volume in first second; GERD, gastroesophageal reflux disease; ICS, inhaled corticosteroids; IL-5, interleukin 5; IQR, interquartile range; na, not available; NSAID, nonsteroidal anti-inflammatory drugs; OCS, oral corticosteroids, methylprednisolone; preBD, pre-bronchodilation.

**Table 2:** Comparison of baseline and follow-up parameters.

	Baseline	Follow-Up	*p*-Value	Baseline	Follow-Up	*p*-Value	*p*-Value
	Responders	Responders	R (Baseline vs. Follow-Up)	Nonresponders	Nonresponders	NR (Baseline vs. Follow-Up)	Follow-Up (R vs. NR)
**Basic characteristics**							
ACT—median (IQR)	15 (8–19)	20.5 (17–24)	0.001	13.5 (7–20)	19 (11–23)	0.375	0.523
**Asthma treatment**							
Regular ICS treatment—n (%)	12 (100)	12 (100)	>0.999	5 (100)	5 (100)	>0.999	0.246
ICS/day (mcg in beclomethasone equivalent)—median (IQR)	600 (450–1050)	700 (400–800)	0.549	600 (440–620)	600 (440–920)	0.440	0.931
OCS maintenance dose—n (%)	3 (25)	0 (0–0)	0.217	5 (100)	5 (100)	0.375	0.0002
OCS maintenance dose (mg/day)—median (IQR)	0 (0–1.5)	0 (0–0)	0.217	12 (5–20)	8 (5–10)	0.468	0.0002
OCS bursts due to asthma exacerbation—median (IQR)	3.5 (2.3–4)	0 (0–0)	<0.0001	5 (2.5–5.5)	1 (1–2.5)	0.040	0.003
**Pulmonary function test**							
FEV1 preBD (mL)—median (IQR)	1770 (1570–2040)	2120 (1673–2498)	0.002	1330 (1010–2265)	2130 (1380–2390)	0.125	0.646
FEV1 preBD (%)—median (IQR)	67.5 (67–80)	81 (74–99)	0.001	46 (36–69)	55 (50–82)	0.125	0.081
**Inflammatory markers**							
Eosinophils in peripheral blood cells (10^6^/L)—median (IQR)	465 (270–690)	65 (40–100)	0.002	790 (170–880)	30 (10–130)	0.032	0.662
Eosinophils in induced sputum (%) *	na	4.5 (2.5–35)	/	na	27 (24–71)	/	0.05
IL-5 in peripheral blood (pg/mL)—median (IQR) **	0.5 (0.3–1.3)	23.8 (13–54.3)	< 0.0001	0.4 (0.1–1.2)	15.8 (10.2–28.4)	0.016	0.442
IL-5 in induced sputum (pg/mL)—median (IQR) ***	na	22.8 (9.2–48.2)	/	na	10.9 (4.8–24.3)	/	0.743

* Only 8 sputa from responder group were representative according to cytology laboratory. ** only sera of 11 responders and 4 non-responders were included. *** one sputum was not included in analysis. Abbreviations: ACT, asthma control test; FEV1, forced expiratory volume in first second; ICS, inhaled corticosteroids; IL-5, interleukin 5; IQR, interquartile range; na, not available; OCS, oral corticosteroids (methylprednisolone); preBD, pre-bronchodilation.

## Data Availability

Data are contained within the article.
